# Statistics of Insanity in England

**Published:** 1890-03-15

**Authors:** 


### Statistics of Insanity in England.

"Statistics of Insanity in England, with Special Reference to Evidence of its Increasing Prevalence," was the title of a paper read by Mr. N. A. Humphreys, of the Registrar-General's Department, at the fourth ordinary meeting of the Royal Statistical Society.

Mr. Humphreys' conclusions may be summed up briefly as follows: 1. It has frequently been admitted by the Lunacy Commissioners that there exists an unknown number of the insane concerning whom they possess no official information, and who are not included in the numbers dealt with in their reports. 2. The census enumerations of 1871 and 1881 throw light upon the amount of this unregistered insanity, since the householder's schedule at each of those censuses registered information concerning all persons of unsound mind enumerated in those years. 3. There is the best ground for believing (a) that these census numbers of the insane err on the ground rather of deficiency than of excess; and (b) that the numbers enumerated in 1881 were more correct; that is, that they were less deficient in that census than at the previous enumeration. 4. The Lunacy Commissioners have frequently expressed the belief that a variety of influences have from time to time in recent years operated to bring within their official cognisance a constantly-increasing proportion of the existing cases of insanity. 5. Assuming the greater accuracy of the census figures as a measure of the aggregate cases of insanity, the increase of existing insanity in the years 1871-81 was 7 per cent. instead of 12 per cent. 6. The proportion to population of cases of insanity known to the Commissioners, although showing a marked and continuous increase during the thirty years embraced by their statistics, also show a marked decline in the rate of increase. 7. The value of the Commissioners' statistics of admissions to asylums is vitiated by the impossibility of separating the really new cases from those admitted from among cases already existing in workhouses, or from pauper cases residing with relatives, or from the large reserve of unregistered cases. 8. Apart from the misconception due to accepting the increasing ratios of registered insanity as conclusive evidence of a real increase of insanity, there has been beyond question an increase in recent years of existing cases of insanity due to what has been called "accumulation." 9. It is shown in the paper that there has been a marked increase in the mean age of inmates of asylums enumerated at each of the last four censuses in 1851, 1861, 1871, and 1881. 10. Insuperable difficulties lie in the way of measuring the effect on the mortality of the insane, and hence upon the existing cases of insanity, of the constant aggregation of increasing proportions of the insane in asylums, because no information exists concerning the rates of mortality among the insane in workhouses, or among those residing with relatives, and neither in asylums or workhouses. Without therefore venturing to say that there has been no increase of insanity in England in recent years, many reasons have been pointed out for hesitation in accepting any insanity statistics which we at present possess as evidence conclusive of any real increase of the rate of occurring insanity.

